# Supplementary material for: Mechanisms governing the pioneering and redistribution capabilities of the non-classical pioneer PU.1
Source: Nat Commun. 2020 Jan 21;11:402. doi: 10.1038/s41467-019-13960-2 (PMC6972792; doi:10.1038/s41467-019-13960-2)
Supplement: Supplementary file 7 — Source data [file 41467_2019_13960_MOESM7_ESM.zip › Source_Data/FigureS2/FigureS2B_MotifScanOutput/KmeansCluster4/homerResults/motif32.similar.html]

motif32

## Information for motif32

A
G
T
C
C
G
A
T
A
G
T
C
G
A
C
T
A
G
T
C
A
C
G
T
A
G
T
C
C
G
A
T
A
G
T
C
A
G
T
C
A
C
G
T
C
T
A
G
A
G
T
C
  
Reverse Opposite:  

C
T
A
G
A
G
T
C
C
G
T
A
A
C
T
G
A
C
T
G
C
G
T
A
C
T
A
G
C
G
T
A
C
T
A
G
C
G
T
A
T
C
A
G
C
G
T
A
C
T
A
G
  

|  |  |
| --- | --- |
| p-value: | 1e-29 |
| log p-value: | -6.893e+01 |
| Information Content per bp: | 1.923 |
| Number of Target Sequences with motif | 61.0 |
| Percentage of Target Sequences with motif | 1.35% |
| Number of Background Sequences with motif | 57.5 |
| Percentage of Background Sequences with motif | 0.14% |
| Average Position of motif in Targets | 96.9 +/- 29.7bp |
| Average Position of motif in Background | 98.6 +/- 59.4bp |
| Strand Bias (log2 ratio + to - strand density) | -0.3 |
| Multiplicity (# of sites on avg that occur together) | 1.00 |
| Motif File: | file (matrix) reverse opposite |

### Similar de novo motifs found

|  |  |  |  |  |  |  |  |
| --- | --- | --- | --- | --- | --- | --- | --- |
| Rank | Match Score | Redundant Motif | P-value | log P-value | % of Targets | % of Background | Motif file |
| 1 | 0.962 | C G A T A G T C A G C T A G T C A C G T A G T C A C G T A G T C A G T C A C G T C T A G A G T C | 1e-22 | -52.406942 | 1.42% | 0.24% | motif file (matrix) |
| 2 | 0.792 | A G T C A C G T A G T C A C G T A G T C C G A T A G C T A G T C A G C T A C T G A G T C | 1e-20 | -46.665353 | 1.86% | 0.48% | motif file (matrix) |
| 3 | 0.844 | A C T G A C G T C T A G T A C G A T G C C G T A C T A G A T C G C G T A T A C G C T G A C A T G C G T A T C A G | 1e-20 | -46.207124 | 1.44% | 0.29% | motif file (matrix) |
| 4 | 0.888 | A C G T A G T C A C G T A G T C A C G T A G T C A G T C A C G T A C T G A G T C | 1e-17 | -39.743200 | 1.88% | 0.56% | motif file (matrix) |
